# Reliability of electronic patient reported outcomes vs. clinical assessment

**DOI:** 10.1192/j.eurpsy.2023.985

**Published:** 2023-07-19

**Authors:** S. Krasteva, Z. Apostolov, H. Kozhuharov

**Affiliations:** Department of Psychiatry and Medical Psychology, Medical University of Varna, Varna, Bulgaria

## Abstract

**Introduction:**

The importance of inter-scale and inter-rater reliability is a well-studied factor in maintenance of data consistency in clinical research. The use of patient reported outcomes poses another risk for compromising data integrity, as some studies show that patients tend to report their symptoms differently in direct clinician-lead interview and self-administered questionnaires. Additionally, as technology is advancing and digital endpoints in CNS clinical trials are becoming a reality, we need to further evaluate if the digital means of self-reporting (e.g., mobile app questionnaires) per se could potentially be a contributing factor in data inconsistency.

**Objectives:**

To assess reliability between clinician-assisted evaluation and electronic patient reported outcomes of depressive and anxiety symptoms.

**Methods:**

Patients not previously diagnosed with depression or anxiety disorders were asked to complete PHQ-9 and/or GAD-7, both verbally administered by a physician. Within 24 hours they were asked to complete a digital form of the same questionnaires.

**Results:**

The analysis of 40 completed double assessments showed no correlation for depressive symptoms presence and severity measured by clinician-lead evaluation and electronic patient reported outcomes (Spearman rho = + 0.191, p=0.686), and poor correlation for anxiety symptoms (Spearman rho = + 0.466, p=0.080).

**Conclusions:**

Many factors interfere with data consistency in clinical research, thus the methods and means of evaluation need to be taken into consideration. The reliability of electronic patient reported outcomes needs to be further assessed and preferably cross-checked by using other validated methods of assessment.

**Disclosure of Interest:**

None Declared

